# Hydration status of underground miners in a temperate Australian region

**DOI:** 10.1186/1471-2458-13-426

**Published:** 2013-05-02

**Authors:** Benjamin G Polkinghorne, Vinodkumar Gopaldasani, Susan Furber, Brian Davies, Victoria M Flood

**Affiliations:** 1 Public Health Officer Training Program, New South Wales Ministry of Health, 73 Miller Street, North Sydney, NSW 2060, Australia; 2 School of Health Sciences, Faculty of Health & Behavioural Sciences, University of Wollongong, Wollongong, NSW 2522, Australia; 3 Health Promotion Service, Illawarra Shoalhaven Local Health District, Building 39B, University of Wollongong, Wollongong, NSW 2500, Australia; 4 School of Public Health and Community Medicine, University of New South Wales, Sydney, NSW, Australia

**Keywords:** Mining, Underground, Dehydration, Hydration

## Abstract

**Background:**

Dehydration is a health risk for miners in tropical regions of Australia. However, it is not known whether dehydration poses a health risk to miners working in temperate regions of Australia.

**Methods:**

A cross-sectional study of 88 miners from two underground mines was undertaken in south-eastern New South Wales, Australia. Participants had their height, weight, waist circumference and hydration status measured and completed a self-administered questionnaire on fluid intake, access to water, and socio-demographic characteristics. Health and Safety managers were surveyed about guidelines relating to healthy work and lifestyle behaviours which impact/influence hydration.

**Results:**

Hydration tests indicated that more than half of the miners (approximately 58%) were dehydrated (Urinary Specific Gravity (USG) >1.020) both before and after their shift, with three workers pre-shift and four workers post-shift displaying clinical dehydration (USG>1.030). Overall, 54.0% of participants were overweight and 36.8% were obese. Miners who commenced the shift with poor hydration status were 2.6 times more likely to end the shift with poor hydration, compared to those who commenced the shift with good hydration (OR 2.6, 95% CI 1.06, 6.44). Miners who had a mean USG result for the entire shift indicating dehydration were more likely to be obese (42.9%) and have a waist measurement in the high risk range for metabolic complications (40.8%) than those workers that were adequately hydrated for their entire shift (29.4% and 14.7% respectively). Some guidelines promoting healthy lifestyles and supportive work environments were in place, but there were limited guidelines on healthy weight and hydration.

**Conclusions:**

Dehydration, being overweight and obesity were linked issues in this cohort of miners. Strategies are needed to: adapt the workplace environment to increase water accessibility; encourage appropriate consumption of water both at work and at home; and to promote physical activity and good nutrition to maintain healthy weight.

## Background

A study of underground miners in northern Australia, in a tropical climate, found that most miners were starting their shifts in a dehydrated state [1]. Kalkowsky and Kampmann studied underground coal miners in Germany and found that only 50-60% of fluid lost in sweat was replaced during the shift [2]. Employees beginning work in a dehydrated state who become further dehydrated during their shift can experience a 4–5% loss in body weight and an increased heart rate of 16–20 beats per minute [3]. There is increasing evidence that mild dehydration may play a role in various morbidities. Good hydration has been shown to reduce the risk of urolithiasis, constipation, exercise asthma and is associated with a reduction in gallstones, dental disease, urinary tract infections, hypertension, fatal coronary heart disease, venous thromboembolism, stroke and chronic renal disease [4,5].

Workplace based interventions to promote health and well-being typically target lifestyle behaviour modification of workers, such as physical activity and dietary interventions [6-8]. Improved access to and provision of, palatable drinking water and removal of energy dense, nutrient poor beverages from the worksite may assist in promoting water consumption, thus maintaining the workers’ hydration status. Consumption of sugary drinks especially caffeinated soft drinks has a diuretic effect due to two mechanisms: osmotic diuresis and caffeine; both of which stimulate the kidneys to release more water [9]. Shift workers can consume up to three servings of soft drink in a single shift [10], which could contribute to diuresis in the absence of sufficient drinking water.

To our knowledge, no studies have been published on the hydration status of miners working in temperate regions. An unpublished pilot investigation on the hydration status of a small sample of miners at one of the mines in the present study found that dehydration was an issue in this cohort [11].

The present study aims to:

1. Determine the hydration status of a cohort of miners in a temperate region of Australia; and

2. Describe the lifestyle health behaviours and management guidelines that affect health and hydration among employees in these mines.

## Methods

Ethics for the study was approved by the University of Wollongong and Illawarra Shoalhaven Local Health District, Health and Medical Human Research Ethics Committee (HE11/312).

### Recruitment of miners

Participants were voluntarily recruited from two underground mines located in the temperate southern district of New South Wales (NSW), Australia after receiving a short briefing on the purposes of the study. All participants were male, aged 18 years and older. Participants conducted diverse tasks in the mine with the majority requiring strenuous physical exertion. The following assumptions were made: Metabolic workload at 200 W/m^2^ (Intense arm and trunk work) and sweat rate not exceeding 1000mL/hr, with acclimatisation. Participants worked at either the long wall or the development panel.

### Data collection

An index of hydration using urine specific gravity (USG) was measured both before and after their shift using a handheld digital optical refractometer (ATAGO® Pocket PAL-10S). Urine samples were tested within an hour of collection for specific gravity only and discarded immediately. This method of determining hydration status of miners has been used previously in both underground and open-cut mines [1,12-14]. To simplify participant feedback, we chose a delineation between adequate and poor hydration based on a USG value of 1.020 which is the cut-off point for adequate hydration favoured by several international sporting associations [15-18]. We used a USG of >1.030 to indicate clinical dehydration, a cut-off which has been used frequently by both industry and sporting bodies [1,14,19,20].

Weight and height were measured to calculate body mass index (BMI). Waist circumference was measured to determine central adiposity. Participants were weighed and measured while clothed in shirts and pants or overalls. Five participants were also weighed and had their height measured while wearing their boots. Their height and weight results were adjusted to account for the weight and sole thickness of a standard work boot.

A self-administered questionnaire collected information on the fluid intake habits of participants at work and at home, participant’s opinions on the adequacy of workplace facilities for water access, their barriers to water consumption, smoking status and socio-demographic characteristics.

### Guidelines on healthy behaviours and supportive environments

Semi-structured interviews were conducted with the Health and Safety Managers of the two mines and the Health and Safety Manager of the parent company responsible for overarching health and safety programs in both sites. The semi-structured interviews were used to identify existing workplace guidelines related to promotion of healthy behaviours and supportive environments, including fluid consumption and access to water. The interviews took approximately 30 minutes each and were conducted separately with each manager.

### Analyses

Total daily liquid nutrient consumption was calculated from participant surveys using the nutritional software package FoodWorks® Pro 2009. Statistical analyses of participant measures were performed using PASW® Statistics 18, release 18.0.0. Chi-square tests were used to examine the differences in categorical variables. Cohen’s kappa coefficient was generated to test the agreement between hydrated and dehydrated status, pre and post shift, and was interpreted according to the guidelines proposed by Landis and Koch [21]: with kappa <0.20 indicating poor agreement; 0.2–0.4 indicating fair agreement; 0.61–0.80 indicating good agreement; and 0.81-1.0 indicating very good agreement. The semi-structured management interviews were summarised in tabular format and results compared between the mines and the parent company.

## Results

In total, 88 miners of a possible 310 were recruited to the study giving a participation rate of 28.4%. All participants worked either a 12 hour weekend day shift or a 12 hour weekend night shift. Testing was conducted over two consecutive weekends in October 2011 (mid-spring in Australia). The monthly mean temperature for the region was 23.1°C (range 16.0-33.6°C).

### Urine specific gravity

Hydration was measured by testing urine specific gravity (USG) both pre-shift and post-shift with 87 participants providing pre-shift urine samples and 84 providing a post-shift sample. Complete pre- and post-shift samples were provided by 83 participants.

**Figure 1:**
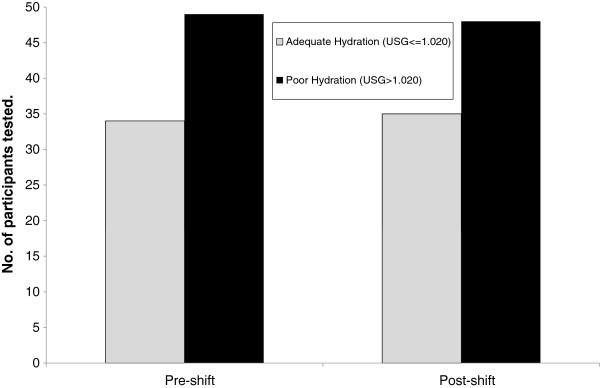
Hydration status of participants at the beginning and end of the shift measured by Urinary Specific Gravity (USG) (n=83).

The mean pre-shift USG was 1.020 (range 1.004-1.033, SD 0.007), and 41% of participants were categorised as hydrated (USG ≤1.020) compared to 59% as dehydrated (USG>1.020). Similarly at the end of the shift, the mean USG was 1.020 (range 1.002-1.034, SD 0.009) with 42% of participants categorised as hydrated compared to 58% as dehydrated (χ^2^=4.44, p=0.035). Three miners demonstrated clinical dehydration (USG >1.030) pre-shift and four miners demonstrated clinical dehydration at post-shift. Figure 1 shows the prevalence of dehydration within the participants at both pre- and post-shift testing.

Further analysis of individual workers' hydration status pre- and post-shift indicated that using Landis and Koch’s guidelines for interpreting Cohen’s kappa coefficient [21], there was “fair” agreement that those participants who were hydrated pre-shift remained hydrated post-shift and vice versa (kappa=0.231, P=0.035). Workers who commenced the shift with poor hydration were 2.6 times more likely to finish the shift poorly hydrated compared to those who commenced the shift with good hydration (Odds Ratio 2.6, 95% CI 1.06 – 6.44).

### Anthropometric and survey measures

To further explore the reasons for dehydration in this cohort a number of anthropometric, demographic and quantitative data were collected from participants via direct measurement and paper based surveys. Not all respondents returned their surveys and of those that did, some selected not to answer certain portions, thus n differs for each category. The results are summarised by the mean hydration status of participants (pre-shift USG + post-shift USG / 2) in Table 1.

**Table 1:** Anthropometric, demographic and behavioural characteristics of workers at two underground mines by mean* hydration status (n=83)

**Measure**	**Category**^**†**^	**Hydrated n (%)**	**Dehydrated n (%)**
**Age (years)**	18-29	9 (26.5)	5 (10.2)
	30-49	17 (50.0)	30 (61.2)
	50-69	5 (14.7)	4 (8.2)
	Incomplete	3 (8.8)	10 (20.4)
**Distance from home to work (km)**	0-39	18 (52.9)	25 (51.0)
	40-99	11 (32.4)	14 (28.6)
	≥100	2 (5.9)	0 (0.0)
	Incomplete	3 (8.8)	10 (20.4)
**Most common drink at work**	Water	29 (85.3)	41 (83.7)
	Other	2 (5.9)	0 (0.0)
	Incomplete	3 (8.8)	8 (16.3)
**Body mass index (kg/m**^**2**^)^**^	Normal	4 (11.8)	4 (8.2)
	Overweight	20 (58.8)	24 (49.0)
	Obese	10 (29.4)	21 (42.9)
**Waist circumference (cm)**^**‡**^	Healthy range	16 (47.1)	17 (34.7)
	Risk range	13 (38.2)	12 (24.5)
	High risk range	5 (14.7)	20 (40.8)

^*^Average of pre-shift and post-shift USG result: Hydrated <=1.020, Dehydrated >1.020.

^†^All category ranges are for adult males.

^**^BMI ranges: Normal (18.50-24.99); Overweight (25.00-29.99); Obese (≥30.00).

^‡^Waist circumference ranges: Healthy range (<94); Risk range (94–102); High risk range (>102).

Table 1 shows that the workers whose mean USG result was greater than 1.020 were more likely to be obese (42.9%) and have a waist measurement in the high risk range for metabolic complications (40.8%) than those workers whose mean USG result was 1.020 or less (29.4% and 14.7% respectively).

Additional survey data indicate that approximately three quarters of respondents believed that they currently drink enough liquid at both work and home to remain adequately hydrated (74.0% and 76.7% respectively). Of these respondents, the majority had a mean USG result >1.020 (62.3% and 58.2% respectively). To increase their actual consumption of water at the workplace 41.8% answered that more reliable access to cold palatable water at the workface, or better underground storage such as large coolers would be preferable. A further 20.0% suggested low calorie flavour additives would increase water palatability and 10.9% suggested that roster changes including additional breaks would increase their water consumption. Alternatively, nearly a third of participants (30.9%) reported that they managed their own hydration adequately and increased their water consumption when working in hot or humid conditions. At the time they completed the survey, participants had not been informed of their measured hydration status.

### Fluid consumption survey

Participants were also asked to record the number of servings of different beverages they had consumed over the preceding month. Table 2 shows the results from the 68 participants who responded by the average of their pre and post-shift hydration status.

**Table 2:** Self-reported mean number of daily fluid servings among participants by mean* hydration status (n=68)

**Hydration status**	**Water (250 mL)**	^**†**^**Tea & coffee (250 mL)**	**Alcohol (Standard drinks)**	^**^Sugary drinks (250-500 mL)	**Milk (250 mL)**	**Other**
Hydrated (n=27)	3.0	1.8	1.5	1.4	1.0	0.1
Dehydrated (n=41)	3.0	1.7	1.6	1.3	1.0	0.2

^*^Average of pre-shift and post-shift USG result.

^†^Coffee represents 71.4% of this total.

^**^Includes fruit juice, soft drinks, sports drinks and energy drinks.

There was almost no difference between average daily consumption of fluids between the hydrated and dehydrated participants (Table 2). Water was the most common beverage consumed, followed by tea, coffee, alcoholic beverages, and high sugar beverages (fruit juice, soft drinks, cordial, energy drinks and sports drinks). The reported consumption of energy and sports drinks was low compared with other sugary beverages.

### Guidelines on lifestyle health behaviours and supportive work environments

A summary of the guidelines for healthy lifestyles and healthy work environments, discussed during the interviews with health and safety managers is provided in Table 3.

**Table 3:** Summary of healthy behaviour and supportive environments guidelines

**Guideline**	**Parent company**	**Mine sites**
**Medical**	3 yearly medical checkups and free access to medical consultant	As per parent company
**Smoking**	Quit Program	Banned on-site
**Alcohol and other drugs**	Education, rehabilitation and deterrent program	As per parent company
**Healthy eating**	Education in training sessions	None
**Exercise**	Participation support program for community based physical activity	As per parent company
**Fatigue Management**	Fatigue management standard, with restrictions on shift duration, breaks and other support.	As per parent company
**Hydration**	None	None
**Heat Stress**	None	Heat Stress TARP^*^ tiered response to high heat and humidity levels. Only applies at one mine.
**Other programs**	Annual health seminars and access to counselling for employees and their families	As per parent company

^*^Trigger Action Response Plan - defines a minimum set of actions required in response to a hazard.

#### General health and safety programs/policies

• *Medical -* An overarching medical program is applicable to both mines and embeds health and surveillance programs at each site; it includes triennial medical examinations and access to a sports medicine specialist for both work related and non-work related medical issues for all workers.

• *Smoking -* Both mines have a total site smoking ban. An overarching Quit Program links miners into government sponsored programs and provides access to counselling services.

• *Alcohol and other drugs -* Both mines have alcohol and other drugs guidelines based on education, rehabilitation and deterrence. Alcohol cannot be brought to or consumed at either site.

• *Fatigue management -* Both sites have a set number of allowable work hours per shift and per week with adequate breaks within and between shifts provided. Transport home and other arrangements are provided when required.

• *Other health and safety programs -* Annual training seminars on topics such as nutrition, hydration, exercise, noise-induced hearing loss, dust inhalation, hazardous substances and chemicals are provided by the health and safety staff or invited experts. All employees and their families also have access to counselling through the Employee Assistance Program.

#### Healthy eating

There are no specific healthy eating guidelines for the mining company or the two mines. Both mines have unregulated vending machines containing energy dense/nutrient poor foods and drinks as there are no canteens onsite or nearby. Miners typically bring their food and drinks, including meals with them to work.

#### Exercise

An overarching corporate guideline applies to both mines whereby they aim to have their workforce engaged annually in a community based physical activity event such as fun runs and team sports. Promotion of activities is provided onsite and entry fees are subsidised.

#### Hydration

Neither mine has guidelines regarding hydration or access to water for miners. Refrigerated water and ice are available on the surface at both mines and supplies of unrefrigerated water are available underground. However, worker access to these supplies varies due to the considerable distance some work sites are from one another. Water supplies are transported to the worksite by vehicle; however there is no set schedule for re-supply.

#### Heat stress

One mine has a heat stress Trigger Action Response Plan (TARP). A TARP defines a minimum set of actions required in response to a hazard, in this case defining limits for heat and humidity in the work area. This TARP describes a tiered response for differing levels of heat and humidity including ensuring miners have sufficient drinking water to prevent dehydration. The amount of water that should be provided or how it will be provided is undefined.

## Discussion

The present study’s findings show that dehydration, being overweight and obesity are issues for this cohort of miners in temperate south-eastern New South Wales.

### Hydration

The pre-shift hydration status of underground mine workers in our study appears to be the most important factor in determining overall hydration status. Consumption of water during a shift is unlikely to rehydrate an already dehydrated worker due to the influence of metabolic work load and sweat rates during the shift. A study of miners in a deep underground coal mine in Germany found that only 50-60% of fluid lost in sweat was replaced during the shift [2].

Our study found that 59% of miners had a USG >1.020 pre-shift and 58% post-shift with most miners who arrived to work dehydrated staying dehydrated and vice versa. A similar study in a deep underground mine in tropical north Australia found that over 60% of participants had a USG >1.022 pre-shift which did not significantly change at end of shift [1]. A study in an open-cut mine in tropical north Australia found that approximately two thirds of workers arrived at work already dehydrated (USG >1.022) [22]. Australian and international evidence suggests that poor hydration is common in mine workers in comparable mines with large water deficits not adequately replaced during recreation times [1,14].

The USG testing in our study involved a hand-held refractometer. This is a simple to use and relatively inexpensive piece of equipment that could be used for self testing and could also potentially be included in the current annual medical checks and alcohol and other drugs testing regimes. Despite being naive to their pre-shift hydration results, anecdotal reports from several of the participants in our study indicated that they had tried to drink extra water during the shift to “improve” on their pre-shift hydration status, thus the act of testing may increase miner’s mindfulness of their hydration status and encourage them to consume more water. The findings from the present study suggested that increased supply of cold palatable water at the workface and provision of flavour additives might increase their consumption of water, consistent with previous research conducted in a fly-in/fly-out mine in tropical north-eastern Australia [14].

### Overweight and obesity

The participants had an average BMI of 29 kg/m^2^ which is at the top end of the overweight range [23] (54.0% overweight and 36.8% obese). These results are comparable with those of two similar studies in the tropical north of Australia who reported an average BMI of 27.9 (range 22.1-38.2) [1] and 29.6 (range 20.2-40.6) [13]. Kalkowsky and Kampmann reported an average BMI of 27.1 [2].

We additionally measured waist circumference to determine central adiposity. A waist measurement for a man in excess of 94cm indicates increased risk of metabolic complications [23] and as both mines exhibited an average waist circumference of 94cm or higher (30.1% in the risk range and 31.3% in the high risk range) therefore, lean body mass is unlikely to explain the high average BMI readings. In 2007–08, 42% of Australian men were overweight and 25% were obese [24] but the rates at our two sites were considerably higher than this. Waist circumference was not recorded in any similar studies.

Importantly, participants in our study with a mean USG indicating poor hydration were more likely to be obese and have a waist measurement in the high risk range. Elevated BMI has also been reported as a significant risk factor for heat exhaustion in mines with that risk increasing as BMI increases [25]. The fluid recall surveys indicated that participants consumed, on average, the same number of fluid servings daily, indicating that fluid intake was not adequately adjusted for physical size. The majority of survey respondents rated their fluid intake as adequate to maintain hydration. A similar study found miners almost exclusively (95%, n=74) perceived their usual fluid intake as adequate despite a group mean USG of 1.022 at both 0600 and 1800 readings [14].

### Alcohol

In our study, participants reported drinking an average of 1.5 standard alcoholic drinks per day which places them within healthy alcohol consumption levels [26,27]. However consumption varied greatly with one participant reporting consumption of an average of approximately 8 standard drinks per day. Alcohol consumption surveys are typically subject to both recall problems and intentional misreporting [28]. We attempted to minimise these biases by providing pre-coded surveys with a combined top value (4 serves per day) and by recording personal details separate to coded survey data.

### Caffeine and energy drinks

Energy drinks have been under scrutiny recently, with reports that between 2003 and 2010, 297 callers to the Australian Poisons Information Centre reporting symptoms of caffeine toxicity due to consumption of energy drinks [29]. There are no current standard guidelines on healthy consumption of caffeine in Australia. The American Medical Association Council on Scientific Affairs considers up to 250 mg of caffeine daily to be an average or moderate amount of caffeine for the average adult [30]. A report by Food Standards Australia New Zealand published in 2000 agrees with this definition [31]. However, the participants in our study reported consuming caffeine on average at only 50-60% of this level. This may be due to the difficulty in accessing caffeinated beverages during work shifts. Miners have free access to tea, coffee and hot chocolate at ground level. Soft drink vending machines are also available, but consumption onsite is limited due to the difficulties in transportation and a total ban for aluminium products underground. Despite some anecdotal concern at both sites about the consumption of energy and sports drinks, neither beverage type was reported to be consumed in high levels.

### Guidelines on lifestyle health behaviours and supportive environments

The parent mining company and the two mines studied employ several guidelines to promote healthy lifestyles and a supportive working environment. However, given our results showing that dehydration and overweight and obesity are significant issues at these two mines, employees may benefit from more education on the benefits of good hydration, good nutrition and increasing physical activity at both the worksite and at home. Smoking and excessive consumption of alcohol and caffeine do not appear to be major additional risk factors for this cohort of industrial miners.

Mine workers typically perform physically demanding tasks over long shifts, thus the primary focus of physical activity promotion programs must be on leisure time. A recent randomized controlled trial of a workplace-based weight loss program in a large Australian aluminium smelter used multiple interventions to increase overall physical activity. Interventions included an information session, program booklets, group-based financial incentives and access to a free online weight loss program. The study reported statistically significant weight loss and reduction of waist circumference compared with controls after 14 weeks [10].

Education is also needed to encourage adequate drinking of water both inside and outside of work hours. Several studies have proposed fluid intake regimes for miners and outdoor workers, [1,12,22] but to our knowledge no follow up studies have been conducted to assess their effectiveness. Our study opens up the possibility of studying specific interventions (environmental and behavioural) targeting ways to improve access to palatable water at the mine sites and encouraging adequate water intake at home and at work.

### Limitations

This was a cross-sectional study of two mines from the same mining company in a temperate region of NSW which could limit the generalisation of the results to other mines in Australia. The BMI and waist circumference results must be interpreted with caution as due to time and access constraints, participants were required to be weighed and measured while clothed. Also, a small study (n=18) indicated that USG was overestimated compared with serum osmolality, for athletes with a high muscle mass, due to increased urine protein metabolites [32]. Therefore, the hydration status of participants with a high BMI must be interpreted with caution. Additionally as mentioned above the participant surveys and fluid frequency records were subject to recall bias and are likely to under-report absolute intake [33].

## Conclusion

Our results show that dehydration is a risk for underground miners working in a temperate zone and that these miners need to improve their hydration between shifts and during shifts. Overweight and obesity are also a significant linked issue in this cohort. These findings suggest the need for health promotion interventions to improve hydration and healthy weight status among miners.

## Competing interests

All authors declare that they have no competing interests.

## Authors’ contributions

BP developed the ethics application, developed and tested the project materials and conducted the testing and data analysis and drafted the manuscript, VG conducted the testing and assisted with developing and testing the project materials and data analysis. VF and SF devised the study and assisted with testing and data analysis. BD assisted with developing and facilitating the study and all authors contributed to developing and revising the manuscript. All authors read and approved the final manuscript.

## Pre-publication history

The pre-publication history for this paper can be accessed here:

http://www.biomedcentral.com/1471-2458/13/426/prepub

## References

[B1] Brake DJ Bates GP Fluid losses and hydration status of industrial workers under thermal stress working extended shifts Occup Environ Med 2003 60 90 96 12554834 10.1136/oem.60.2.90 PMC1740457

[B2] Kalkowsky B Kampmann B Physiological strain of miners at hot working places in German coal mines Ind Health 2006 44 465 473 16922191 10.2486/indhealth.44.465

[B3] Kenefick RW Sawka MN Hydration at the work site J Am Coll Nutr 2007 26 Suppl 5 597 603 10.1080/07315724.2007.10719665 17921472

[B4] Manz F Hydration and Disease J Am Coll Nutr 2007 26 Suppl 5 535 541 10.1080/07315724.2007.10719655 17921462

[B5] Strippoli GF Craig JC Rochtchina E Flood VM Wang JJ Mitchell P Strippoli GF, Craig JC, Rochtchina E, Flood VM, W Fluid and nutrient intake and risk of chronic kidney disease Nephrology (Carlton) 2011 16 326 334 21342326 10.1111/j.1440-1797.2010.01415.x

[B6] Anderson LM Quinn TA Glanz K Ramirez G Kahwati LC Johnson DB Buchanan LR Archer WR Chattopadhyay S Kalra GP The Effectiveness of Worksite Nutrition and Physical Activity Interventions for Controlling Employee Overweight and Obesity: A Systematic Review Am J Prev Med 2009 37 340 357 19765507 10.1016/j.amepre.2009.07.003

[B7] Conn VS Hafdahl AR Cooper PS Brown LM Lusk SL Meta-analysis of workplace physical activity interventions Am J Prev Med 2009 37 330 339 19765506 10.1016/j.amepre.2009.06.008 PMC2758638

[B8] Groeneveld IF Proper KI van der Beek AJ Hildebrandt VH van Mechelen W Lifestyle-focused interventions at the workplace to reduce the risk of cardiovascular disease–a systematic review Scand J Work Environ Health 2010 36 202 215 20066386 10.5271/sjweh.2891

[B9] Mudge DW Johnson DW Coca-Cola and kangaroos Lancet 2004 364 1190 1190 15451232 10.1016/S0140-6736(04)17111-0

[B10] Morgan PJ Collins CE Plotnikoff RC Cook AT Berthon B Mitchell S Callister R Efficacy of a workplace-based weight loss program for overweight male shift workers: The Workplace POWER (Preventing Obesity Without Eating like a Rabbit) randomized controlled trial Prev Med 2011 52 317 325 21300083 10.1016/j.ypmed.2011.01.031

[B11] Hines J Heat Stress in the Development Panel MSc Research Project Report 2011 Wollongong: University of Wollongong, Occupational Health and Safety Department

[B12] Miller V Bates G Hydration of outdoor workers in north-west Australia J Occup Health Safety Aust NZ 2007 23 79 87

[B13] Donoghue AM Sinclair MJ Bates GP Heat Exhaustion in a Deep Underground Metalliferous Mine Occup Environ Med 2000 57 165 174 10810098 10.1136/oem.57.3.165 PMC1739920

[B14] Carter A Muller R Hydration knowledge, behaviours and status of staff at the residential camp of a fly-in/fly-out minerals extraction and processing operation in tropical North-Eastern Australia Ind Health 2007 45 579 589 17878630 10.2486/indhealth.45.579

[B15] Armstrong LE Pumerantz AC Fiala KA Roti MW Kavouras SA Casa DJ Maresh CM Human hydration indices: acute and longitudinal reference values Int J Sport Nutr Exerc Metab 2010 20 145 153 20479488 10.1123/ijsnem.20.2.145

[B16] American College of Sports Medicine Position Stand – Exercise and Fluid Replacement Med Sci Sports Exerc 2007 39 377 390 17277604 10.1249/mss.0b013e31802ca597

[B17] Casa DJ Armstrong LE Hillman SK Montain SJ Reiff RV Rich BS Roberts WO Stone JA National Athletic Trainers’ Association Position Statement: Fluid replacement for athletes J Athl Train 2000 35 212 224 16558633 PMC1323420

[B18] Osterberg KL Horswill CA Baker LB Pregame Urine Specific Gravity and Fluid Intake by National Basketball Association Players During Competition J Athl Train 2009 44 53 57 19180219 10.4085/1062-6050-44.1.53 PMC2629040

[B19] Kavouras SA Assessing hydration status Curr Opin Clin Nutr Metab Care 2002 5 519 524 12172475 10.1097/00075197-200209000-00010

[B20] Oppliger RA Magnes SA Popowski LA Gisolfi CV Accuracy of Urine Specific Gravity and Osmolality as Indicators of Hydration Status Int J Sport Nutr Exerc Metab 2005 15 236 251 16131695 10.1123/ijsnem.15.3.236

[B21] Landis JR Koch GG The measurement of observer agreement for categorical data Biometrics 1977 33 159 174 843571

[B22] Carter A Muller R Roberts S The hydration status and needs of workers at a north-west Queensland fertilizer plant J Occup Health Safety Aust NZ 2006 22 73 82

[B23] WHO Consultation on Obesity Obesity - preventing and managing the global epidemic: report of a WHO consultation World Health Organ Rep Tech Rep Ser 2000 894 1 253 11234459

[B24] Australian Bureau of Statistics 4842.0.55.001 - Overweight and Obesity in Adults in Australia: A Snapshot, 2007–08 2011 Australia: ABS. Canberra

[B25] Donoghue AM Bates GP The risk of heat exhaustion at a deep underground metalliferous mine in relation to body-mass index and predicted *V*O_2_max Occup Med 2000 50 259 263 10.1093/occmed/50.4.259 10912377

[B26] National Health and Medical Research Council Australian guidelines to reduce health risks from drinking alcohol 2009 Australia: NHMRC. Canberra

[B27] Australian Institute of Health and Welfare Drugs in Australia 2010: tobacco, alcohol and other drugs Volume Drug statistics series no. 27 2011 Australia: AIHW. Canberra

[B28] Dawson DA Methodological issues in measuring alcohol use Alcohol Res Health 2003 27 18 29 15301397 PMC6676704

[B29] Gunja N Brown J Energy drinks: health risks and toxicity Med J Aust 2012 196 46 49 22256934 10.5694/mja11.10838

[B30] American Medical Association Council on Scientific Affairs Caffeine Labeling JAMA 1984 252 803 806 6748182

[B31] Smith P Smith A Miners J McNeill J Proudfoot A Report from the expert working group on the safety aspects of dietary caffeine 2000 Canberra, Australia: ANZFA

[B32] Hamouti N Coso J Ávila A Mora-Rodriguez R Effects of athletes’ muscle mass on urinary markers of hydration status Eur J Appl Physiol 2010 109 213 219 20058021 10.1007/s00421-009-1333-x

[B33] Riley M Rutishauser IHE Webb K Comparison of short questions with weighed dietary records 2001 Canberra, Australia: Australian Food and Nutrition Monitoring Unit

